# Next-generation sequencing of primary testicular lymphoma and relapse in the glans penis after prophylactic radiation therapy: a rare case report

**DOI:** 10.1186/s13000-024-01498-x

**Published:** 2024-06-03

**Authors:** Naoya Ishibashi, Yoko Nakanishi, Toshiya Maebayashi, Katsuhiro Miura, Sumie Ohni, Shinobu Masuda, Yasuo Amano, Masahiro Okada

**Affiliations:** 1 Department of Radiology, Nihon University School of Medicine, 30-1, Oyaguchi Kami-cho, Itabashi, 173-8610 Tokyo Japan; 2 Department of Oncologic Pathology, Nihon University School of Medicine, Itabashi, 173-8610 Tokyo Japan; 3 Division of Hematology and Rheumatology, Department of Medicine, Nihon University School of Medicine, Itabashi, 173-8610 Tokyo Japan; 4 Department of Radiology, Nihon University Hospital, Chiyoda, Tokyo, 101-8309 Japan

**Keywords:** Next-generation sequencing, Primary testicular lymphoma, Glans penis, Radiation therapy, Case report

## Abstract

**Background:**

Primary testicular lymphoma (PTL) is relatively rare. The contralateral testis is a common site of PTL relapse; therefore, once complete remission is achieved, radiation therapy (RT) is administered to the contralateral testis to prevent relapse.

**Case presentation:**

A 76-year-old man was diagnosed with PTL and received RT as described above. However, despite achieving and maintaining complete remission, a mass diagnosed as diffuse large B-cell lymphoma by tissue biopsy developed in the glans penis 6.5 years after prophylactic RT. We investigated whether the glans penile lymphoma was PTL relapse or a new malignancy by genomic analysis using next-generation sequencing of DNA extracted from two histopathological specimens.

**Conclusions:**

We found the same variant allele fraction in four somatic genes (*MYD88*, *IL7R*, *BLNK*, and *FLT3*) at similar frequencies, indicating that the glans penile lymphoma had the same origin as the PTL. To the best of our knowledge, this is the first case report of PTL relapse in the glans penis.

**Supplementary Information:**

The online version contains supplementary material available at 10.1186/s13000-024-01498-x.

## Background

Primary testicular lymphoma (PTL) is a rare disease, accounting for 1–2% of all non-Hodgkin’s lymphomas [1, 2]. High orchiectomy of the affected testis is performed to attain the diagnosis, and systemic chemotherapy, such as rituximab, cyclophosphamide, doxorubicin, vincristine, and prednisone (R-CHOP), is administered after surgery. After complete remission has been achieved, radiation therapy (RT) is administered to the contralateral testis to prevent relapse because the contralateral testis is a common site of PTL relapse [1, 3, 4]. Prophylactic RT significantly reduces the relapse rate in the contralateral testis [1, 5]. The central nervous system (CNS) is also a common site of PTL relapse [1–4, 6].

We conducted a comprehensive genomic analysis by targeting the exons of oncogenes and tumor suppressor genes via next-generation sequencing (NGS) of DNA extracted from histopathological samples both at the initial diagnosis and at relapse in the glans penis. We hypothesized that if exactly the same variant allele fraction is detected in some somatic genes at similar frequencies in the two histopathological samples, then the glans penile lymphoma shares the same origin as the initial diffuse large B-cell lymphoma (DLBCL) in the testis, confirming the diagnosis of relapsed PTL.

## Case report

**Fig. 1:**
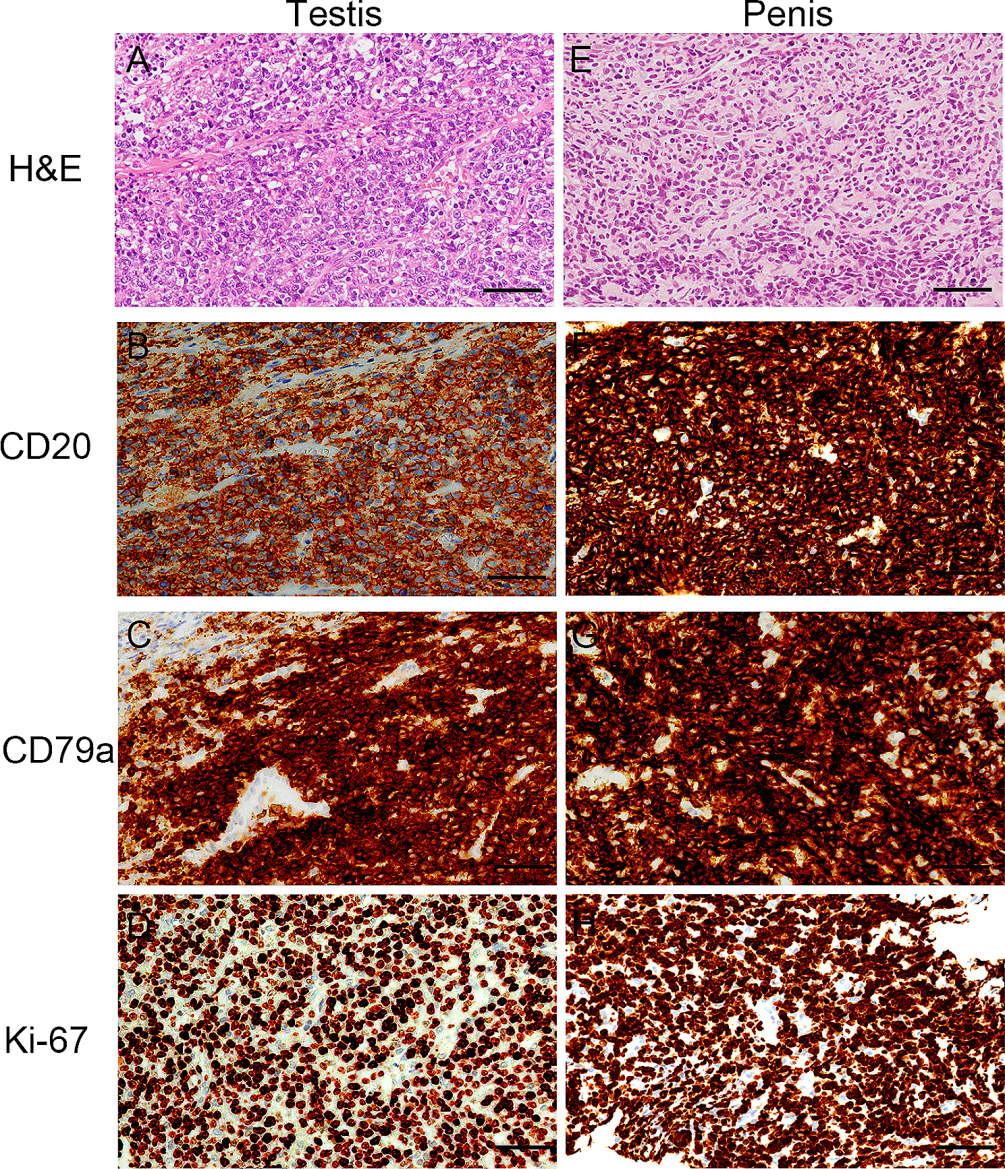
Histological and immunohistochemical features of the (**A**–**D**) testicular and (**E**–**H**) penile samples. Hematoxylin and eosin staining revealed (A) diffuse infiltration of large atypical lymphocytes in the testicular sample and (E) diffuse atypical lymphocytes in the penile sample. The tumor cells in the testicular and penile tissues exhibited diffuse monoclonal expression of (**B**, **F**) CD20 and (**C**, **G**) CD79a. (**D**, **H**) The Ki-67 index was approximately 70–75% in both samples. Each bar represents 50 μm

**Fig. 2:**
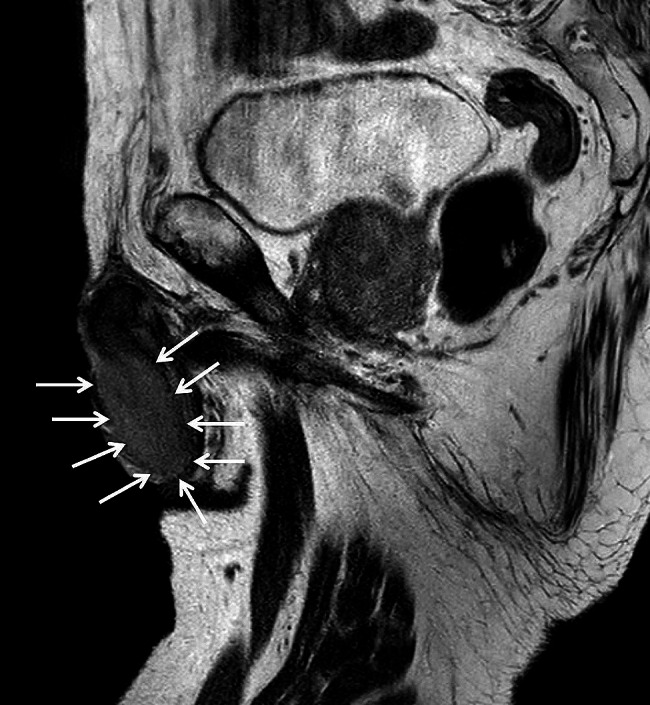
Magnetic resonance imaging of the glans penile tumor. A T2-weighted image revealed a large soft tissue mass in the glans penis and corpus cavernosum (white arrows)

**Fig. 3:**
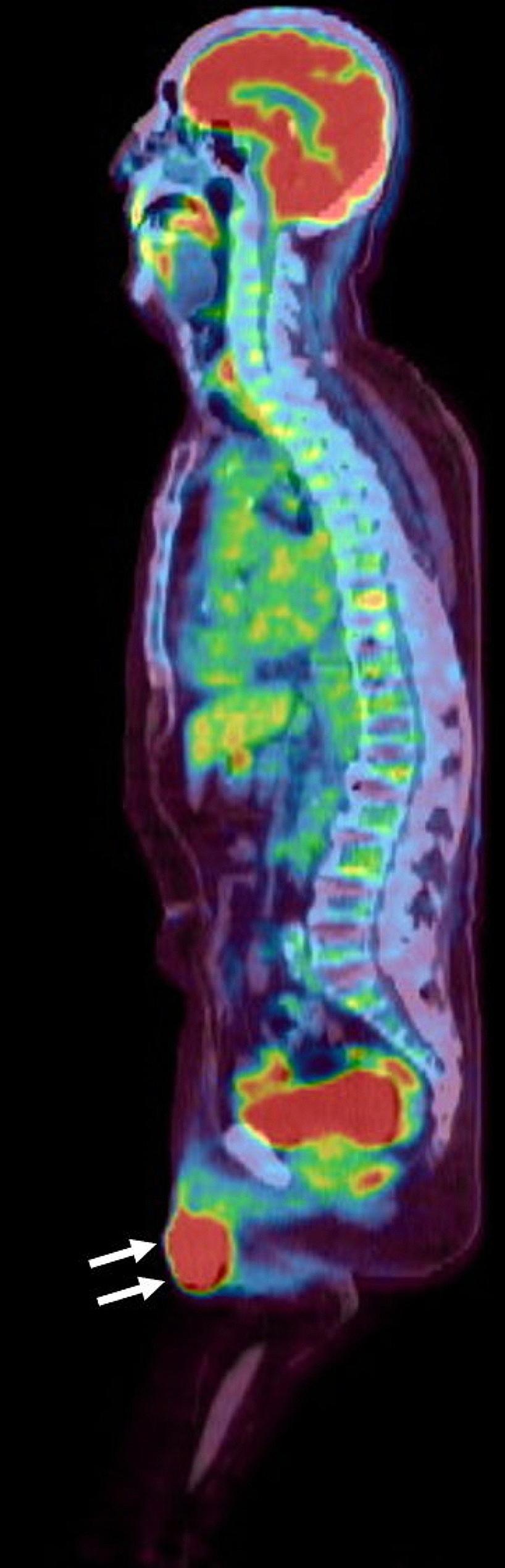
Sagittal fused fluorodeoxyglucose positron emission tomography/computed tomography image. High fluorodeoxyglucose uptake in the glans penile tumor was confirmed (white arrows; maximum standardized uptake value, 33.3)

A 76-year-old Japanese man visited our hospital with painless swelling of the right testis. He underwent high orchiectomy for diagnostic purposes. Pathologic examination revealed marked infiltration of atypical lymphocytes extending from the testis to the epididymis, and immunostaining revealed monoclonal proliferation of B cells positive for CD20 and CD79a. These findings led to a diagnosis of DLBCL (Fig. 1A–D). No infiltration of any other organs was detected by either fluorodeoxyglucose positron emission tomography/computed tomography or bone marrow biopsy. Thereafter, the final diagnosis of the primary testicular DLBCL, Ann Arbor stage I_E_, was confirmed. The patient’s serum lactate dehydrogenase concentration was elevated, and his International Prognostic Index score was 2 points [7]. According to this score, he was deemed at low/intermediate risk and received three cycles of the R-CHOP regimen. During R-CHOP therapy, he also received three doses of intrathecal methotrexate at 15 mg for CNS prophylaxis. The penis was lifted and taped to the abdominal wall for exclusion from the irradiation field, and prophylactic RT was delivered to the contralateral testis with an 8-MeV electron beam at a dose of 2 Gy/fraction to a total dose of 30 Gy. Although the patient remained in complete remission after RT, a mass developed in the glans penis 6.5 years after prophylactic RT. The mass grew, and the entire glans penis was swollen. T2-weighted magnetic resonance imaging revealed a large soft tissue mass from the glans penis to the corpus cavernosum (Fig. 2). Tissue biopsy of the mass in the glans penis revealed marked infiltration of atypical lymphocytes, and immunostaining revealed monoclonal proliferation of CD20- and CD79a-positive B cells. These findings led to a diagnosis of DLBCL (Fig. 1E–H). Fluorodeoxyglucose positron emission tomography/computed tomography revealed infiltration of the glans penis (Fig. 3) and right inguinal lymph node. Magnetic resonance imaging revealed no infiltration of the CNS or contralateral testis. Because the pain in the glans penis was severe, palliative RT was prioritized over systemic therapy. Three-dimensional conformal RT was delivered to the entire penis and right inguinal lymph node using 6-MV photon beams at a dose of 2 Gy/fraction to a total dose of 40 Gy. The glans penile lymphoma markedly decreased in size after RT. A urinary catheter was placed after RT because the patient exhibited grade 2 urinary retention according to the National Cancer Institute Common Terminology Criteria for Adverse Events (CTCAE) v5.0 [8]. Because relapsed DLBCL was considered likely, second-line chemotherapy with polatuzumab vedotin plus bendamustine and rituximab (Pola-BR) was initiated after RT [9]. CTCAE grade 4 skin rashes subsequently appeared, prompting treatment discontinuation after two cycles of Pola-BR. Computed tomography revealed disappearance of the lesion 199 days after RT, and no relapse was observed up to 549 days after treatment completion.

### Next-generation sequencing

The site of the relapse in this patient, the glans penis, is an extremely rare site for relapse of PTL and was located close to the primary radiation field in this case. Additionally, a 6.5-year progression-free period is unusual for aggressive lymphomas. Thus, to distinguish relapse of PTL or secondary DLBCL, we performed a comprehensive genomic analysis using NGS of DNA extracted from both histopathological specimens obtained at the initial diagnosis and at relapse after obtaining written informed consent from the patient.

DNA was extracted from both the initial PTL and glans penile lymphoma specimens. NGS was conducted using the Ion AmpliSeq™ Comprehensive Cancer Panel on the Ion Torrent platform (Thermo Fisher Scientific, Waltham, MA, USA). The Ion AmpliSeq™ Comprehensive Cancer Panel provides complete exon coverage of more than 400 cancer-associated genes. The same variant allele fraction in four somatic genes, namely *MYD88*, *IL7R*, *BLNK*, and *FLT3*, was detected in both histopathological specimens at similar frequencies (37.8–54.9%) (Table 1). Therefore, the glans penile lymphoma was found to share the same origin as the PTL in this patient, leading to a diagnosis of relapsed PTL. Although these genes are not oncogenic, mutations of these genes have been detected in patients with DLBCL and leukemia [10–14]. In particular, *MYD88* encodes for an adaptor protein that mediates Toll-like receptor and interleukin-1 receptor signaling, and mutations in this gene are reportedly important drivers of lymphomagenesis in PTL [11].

**Table 1:** List of common variant allele fraction in testicular and glans penile genomics

Gene	Chr	Locus	RefSeq	Location	Protein	Function	Interpretation	VAF (%)	
								Testicular sample	Glans penile sample
*MYD88*	3	38,182,641	NM_002468.5	c.818T > C	p.L273P	Missense	Uncertain significance	39.1	41.2
*IL7R*	5	35,874,575	NM_002185.5	c.731 C > T	p.T244I	Missense	Benign	48.5	46.7
*BLNK*	10	97,990,583	NM_013314.4	c.171T > C	p.P57=	Silent	Benign	46.2	47.1
*FLT3*	13	28,608,459	NM_004119.3	c.1683 A > G	p.L561=	Silent	Benign	37.8	54.9

Abbreviations: RefSeq = NCBI Reference Sequence Database; VAF = variant allele fraction.

## Discussion

PTL is an aggressive extranodal non-Hodgkin’s lymphoma that relapses in various sites at a high frequency of 52.0–80.0% even after first-line therapy [1–3]. Common extranodal sites of PTL relapse include the CNS and contralateral testis [1–3]. The relapse rate in the contralateral testis is 15% at 3 years without prophylactic RT; however, RT significantly reduces the relapse rate [1]. One study showed that the relapse rate in the contralateral testis was 10% after prophylactic RT [4], whereas another study showed that no relapse was observed in any patients who received prophylactic RT [5]. Thus, the National Comprehensive Cancer Network Clinical Practice Guidelines in Oncology and the International Lymphoma Radiation Oncology Group recommend that prophylactic RT should be delivered to the contralateral testis after chemotherapy regimens such as R-CHOP [15, 16]. Although prophylactic RT is the standard of care for PTL, statistical data from the US Surveillance, Epidemiology, and End Results program showed that only 35.9% of patients received RT [17]. However, prophylactic RT to the contralateral testis has also been reported to improve overall survival [4, 17]. Although other extranodal sites of relapse reportedly include Waldeyer’s ring and the skin, no report has described relapse in the glans penis [1, 3, 4, 6]. A rare case of bilateral synchronous testicular involvement of PTL was recently reported, but the spermatic cord and glans penis were intact by surgical examination [18]. Also in our patient, no infiltration of the spermatic cord by surgical examination at initial high orchiectomy. PTL relapse after first-line therapy occurs relatively early, at a median of 0.8 to 3.0 years. However, relapse in the contralateral testis tends to occur at a median of 45 months after treatment [3, 4]. In our patient, PTL relapsed in the glans penis, but not the contralateral testis, 6.5 years after prophylactic RT. In general, the contralateral testis, CNS and skin of the PTL patient should be evaluated carefully for the management of undiagnosed relapse. But we may also need to evaluate the penis henceforth considering our case. Primary penile lymphoma is extremely rare; approximately 30 cases have been reported to date. Although the use of chemotherapy, RT, surgery, or their combination has been reported, no standard of care has been established for primary penile lymphoma [19–22]. In our patient, palliative RT was prioritized over systemic therapy because of severe pain in the glans penis, and the glans penile lymphoma lesion markedly decreased in size.

A commonly reported histological subtype of primary penile lymphoma is DLBCL [19, 21, 22]. Because both the PTL and glans penile lymphoma in our patient were histologically diagnosed as DLBCL, we performed a comprehensive genomic analysis by targeting the exons of oncogenes and tumor suppressor genes via NGS of DNA extracted from two histopathological samples to determine whether the glans penile lymphoma shared the same origin as the PTL in this patient or whether it was a new primary lesion such as radiation-induced secondary malignancy. Given the reported association between exposure to radiofrequency waves and testicular cancer [23], we were concerned about.

unexpected exposure of the glans penis to a very low dose of radiation from the previously prophylactic RT of the contralateral testis. Although the penis was lifted and taped to the abdominal wall for exclusion from the irradiation field, the corpus spongiosum and cavernosum might have been included the irradiation field. We used small electron cones, and the RT dose in these tissues continuous with the glans penis could not be precisely determined. Because identical mutations in four somatic genes were detected from the two histopathological specimens at similar frequencies, the glans penile lymphoma was found to share the same origin as the PTL, leading to a diagnosis of relapsed PTL. In high-grade BCL, gene expressions such as *MYC* and *BCL-6* alterations have been widely known to be associated with poor prognosis and these specific expression patterns were investigated in PTL [24, 25]. But in our patient, a comprehensive genomic analysis including *MYC* and *BCL-6* extracted from both histopathological specimens revealed no alterations in *MYC* and *BCL-6*.

## Conclusion

In summary, lymphoma developed in the glans penis after a long period following prophylactic RT for PTL. A comprehensive genomic analysis using NGS allowed us to definitively eliminate the possibility of radiation-induced secondary malignancy. To the best of our knowledge, this is the first case report of an NGS-confirmed relapse of PTL in the glans penis.

### Electronic supplementary material

Below is the link to the electronic supplementary material.

Supplementary Material 1

## Data Availability

The dataset used during this study are available from the corresponding author on reasonable request.

## Abbreviations

PTL: Primary testicular lymphoma
R-CHOP: Rituximab, cyclophosphamide, doxorubicin, vincristine, and prednisone
RT: Radiation therapy
CNS: Central nervous system
NGS: Next-generation sequencing
DLBCL: Diffuse large B-cell lymphoma
CTCAE: Common Terminology Criteria for Adverse Events
Pola-BR: Polatuzumab vedotin plus bendamustine and rituximab

## References

[1] Zucca E, Conconi A, Mughal TI, Sarris AH, Seymour JF, Vitolo U, Klasa R, Ozsahin M, Mead GM, Gianni MA, International Extranodal Lymphoma Study Group (2003). Patterns of outcome and prognostic factors in primary large-cell lymphoma of the testis in a survey by the International Extranodal Lymphoma Study Group. J Clin Oncol.

[2] Hasselblom S, Ridell B, Wedel H, Norrby K, Sender Baum M, Ekman T (2004). Testicular lymphoma–a retrospective, population-based, clinical and immunohistochemical study. Acta Oncol.

[3] Fonseca R, Habermann TM, Colgan JP, O’Neill BP, White WL, Witzig TE, Egan KS, Martenson JA, Burgart LJ, Inwards DJ (2000). Testicular lymphoma is associated with a high incidence of extranodal recurrence. Cancer.

[4] Visco C, Medeiros LJ, Mesina OM, Rodriguez MA, Hagemeister FB, McLaughlin P, Romaguera JE, Cabanillas F, Sarris AH (2001). Non-hodgkin’s lymphoma affecting the testis: is it curable with doxorubicin-based therapy?. Clin Lymphoma.

[5] Vitolo U, Chiappella A, Ferreri AJ, Martelli M, Baldi I, Balzarotti M, Bottelli C, Conconi A, Gomez H, Lopez-Guillermo A (2011). First-line treatment for primary testicular diffuse large B-cell lymphoma with rituximab-CHOP, CNS prophylaxis, and contralateral testis irradiation: final results of an international phase II trial. J Clin Oncol.

[6] Cheah CY, Wirth A, Seymour JF, Blood (2014). Prim Testicular Lymphoma.

[7] International Non-Hodgkin’s Lymphoma Prognostic Factors Project (1993). A predictive model for aggressive non-hodgkin’s lymphoma. N Engl J Med.

[8] National Cancer Institute Common. Terminology Criteria for Adverse Events (CTCAE) 5.0. Nov 27, 2017.

[9] Sehn LH, Herrera AF, Flowers CR, Kamdar MK, McMillan A, Hertzberg M, Assouline S, Kim TM, Kim WS, Ozcan M (2020). Polatuzumab Vedotin in relapsed or refractory diffuse large B-Cell lymphoma. J Clin Oncol.

[10] Ngo VN, Young RM, Schmitz R, Jhavar S, Xiao W, Lim KH, Kohlhammer H, Xu W, Yang Y, Zhao H (2011). Oncogenically active MYD88 mutations in human lymphoma. Nature.

[11] Kraan W, van Keimpema M, HorliMYD HM, Schilder-Tol EJ, Oud ME, Noorduyn LA, Kluin PM, Kersten MJ, Spaargaren M, Pals ST (2014). High prevalence of oncogenic MYD88 and CD79B mutations in primary testicular diffuse large B-cell lymphoma. Leukemia.

[12] Oliveira ML, Veloso A, Garcia EG, Iyer S, Pereira C, Barreto VM, Langenau DM, Barata JT (2022). Mutant IL7R collaborates with MYC to induce T-cell acute lymphoblastic leukemia. Leukemia.

[13] Fouquet G, Rossignol J, Ricard L, Guillem F, Couronné L, Asnafi V, Vavasseur M, Parisot M, Garcelon N, Rieux-Laucat F (2022). BLNK mutation associated with T-cell LGL leukemia and autoimmune diseases: Case report in hematology. Front Med (Lausanne).

[14] Perl AE, Larson RA, Podoltsev NA, Strickland S, Wang ES, Atallah E, Schiller GJ, Martinelli G, Neubauer A, Sierra J, et al. Outcomes in patients with FLT3-Mutated Relapsed/ refractory Acute myelogenous Leukemia who underwent transplantation in the phase 3 ADMIRAL trial of Gilteritinib versus Salvage Chemotherapy. Transpl Cell Ther. 2022;22S2666–6367. 10.1016/j.jtct.2022.12.006. 10.1016/j.jtct.2022.12.006 PMC10189888 36526260

[15] NCCN Clinical Practice. Guidelines in Oncology (NCCN Guidelines®) Version 2.2015 Non-Hodgkin’s Lymphomas.

[16] Yahalom J, Illidge T, Specht L, Hoppe RT, Li YX, Tsang R, Wirth A, International Lymphoma Radiation Oncology Group (2015). Modern radiation therapy for extranodal lymphomas: field and dose guidelines from the International Lymphoma Radiation Oncology Group. Int J Radiat Oncol Biol Phys.

[17] Gundrum JD, Mathiason MA, Moore DB, Go RS (2009). Primary testicular diffuse large B-cell lymphoma: a population-based study on the incidence, natural history, and survival comparison with primary nodal counterpart before and after the introduction of Rituximab. J Clin Oncol.

[18] Di Domenico D, Barone B, Del Biondo D, Napolitano L, Fusco GM, Cirillo L, Reccia P, De Luca L, Zito AR, Napodano G (2022). Abnormal presentation of a bilateral, synchronous and plurimetastatic medium and large cell testicular lymphoma: a case report. Mol Clin Oncol.

[19] Arena F, di Stefano C, Peracchia G, Barbieri A, Cortellini P (2001). Primary lymphoma of the penis: diagnosis and treatment. Eur Urol.

[20] McNab PM, Jukic DM, Mills O, Browarsky I (2011). Primary cutaneous CD30 + T-cell lymphoproliferative disorder presenting as paraphimosis: a case report and review of the literature. Dermatol Online J.

[21] Hamamoto S, Tozawa K, Nishio H, Kawai N, Kohri K (2012). Successful treatment of primary malignant lymphoma of the penis by organ-preserving rituximab-containing chemotherapy. Int J Clin Oncol.

[22] Stamatiou K, Pierris N. Lymphoma presenting as cancer of the glans penis: a case report. Case Rep Pathol. 2012;2012(948352). 10.1155/2012/948352. 10.1155/2012/948352 PMC3465891 23056979

[23] Yazici S, Del Biondo D, Napodano G, Grillo M, Calace FP, Prezioso D, Crocetto F, Barone B. Risk factors for testicular Cancer: Environment, genes and Infections-Is it all? Medicina (Kaunas). 2023; 59(4): 724. 10.3390/medicina59040724. 10.3390/medicina59040724 PMC10145700 37109682

[24] Wang Q, Zheng D, Chai D, Wu S, Wang X, Chen S, Wu L, Cao R, Tao Y (2020). Primary testicular diffuse large B-cell lymphoma: Case series. Med (Baltim).

[25] Bruehl FK, Ketterling RP, Rimsza LM, Santos EF, McPhail ED, Bruehl FK, Ketterling RP, Rimsza LM, Santos EF, McPhail ED (2023). J Hematop.

